# Transcriptomics Analysis of the Chinese Pear Pathotype of *Alternaria alternata* Gives Insights into Novel Mechanisms of HSAF Antifungal Activities

**DOI:** 10.3390/ijms19071841

**Published:** 2018-06-22

**Authors:** Feng He, Bingxin Li, Gan Ai, Alex Machio Kange, Yancun Zhao, Xiong Zhang, Yifan Jia, Daolong Dou, Fengquan Liu, Haiqun Cao

**Affiliations:** 1 Institute of Plant Protection, Jiangsu Academy of Agricultural Sciences, Jiangsu Key Laboratory for Food Quality and Safety-State Key Laboratory Cultivation Base of Ministry of Science and Technology, Nanjing 210014, China; mengzhonghefeng_20@163.com (F.H.); lbx525@163.com (B.L.); zhaoyc27@126.com (Y.Z.); yifanjia123@163.com (Y.J.); 2 College of Plant Protection, Anhui Agricultural University, Hefei 230036, China; 3 Department of Plant Pathology, Nanjing Agricultural University, Nanjing 210095, China; 2016102010@njau.edu.cn (G.A.); machiopj@gmail.com (A.M.K.); 2014202002@njau.edu.cn (X.Z.); ddou@njau.edu.cn (D.D.)

**Keywords:** *Alternaria alternata*, signaling pathway, metabolism, HSAF, antifungal mechanism

## Abstract

*Alternaria alternata* (Fries) Keissler is a lethal pear pathogen that causes leaf black spot disease of pear in Southern China. Heat-stable activity factor (HSAF) is a polycyclic tetramate macrolactam (PTM) produced by *Lysobacter enzymogenes* and many other microbes with a broad-spectrum antifungal activity against many filamentous fungi. In this study, we evaluated the antifungal effect of HSAF against *A. alternata* and proposed its antifungal mechanism in *A. alternata*. We report that HSAF inhibited the mycelial growth of *A. alternata* in a dose-dependent manner. Transcriptomics analysis revealed that HSAF treatment resulted in an expression alteration of a wide range of genes, with 3729 genes being up-regulated, and 3640 genes being down-regulated. Furthermore, we observed that HSAF treatment disrupted multiple signaling networks and essential cellular metabolisms in *A. alternata*, including the AMPK signaling pathway, sphingolipid metabolism and signaling pathway, carbon metabolism and the TCA (tricarboxylic acid) cycle, cell cycle, nitrogen metabolism, cell wall synthesis and a key hub protein phosphatase 2A (PP2A). These observations suggest that HSAF breaches metabolism networks and ultimately induces increased thickness of the cell wall and apoptosis in *A. alternata.* The improved understanding of the antifungal mechanism of HSAF against filamentous fungi will aid in the future identification of the direct interaction target of HSAF and development of HSAF as a novel bio-fungicide.

## 1. Introduction

*Lysobacter* spp. belong to the *Xanthomonadaceae* family and are ubiquitous in agricultural environment including soil, water, and the rhizosphere of plants [1]. Many species of *Lysobacter* co-reside in the same environment with plant-associated fungi and, constantly interacting and competing with these fungi for nutrients and space, the antifungal activities are developed. One of them, *Lysobacter enzymogenes*, is known for its strongly proteolytic activity and ability to lyse many filamentous fungi and nematodes. Several strains of *L. enzymogenes*, such as isolate 3.1T8 from the rhizosphere of cucumber [2], isolate C3 from grass foliage [3] and isolate OH11 from the rhizosphere soil of green pepper plants [4], have been developed into biocontrol agents and used to control various plant diseases [2,4,5,6,7,8].

*Lysobacter* could adhere and invade the hyphae of fungal and oomycete pathogens [9]. Moreover, they can produce metabolites that may be a toxin to fungi and oomycete. One of the well-known metabolites HSAF *(heat-stable antifungal factor) is produced by L. enzymogenes* strains C3 and OH11 which plays key roles for adhesion and invasion of *Lysobacter* [10]. The studies further determined that HSAF possesses antifungal activities against numerous phytopathogens, including *Candida albicans, Aspergillus nidulans, Phytophthora capsici*, *Sclerotinia sclerotiorum*, *Fusarium oxysporium*, *Magnaporthe oryzae* and *F. graminearum* [4,11,12]. The antifungal mechanisms of HSAF were also revealed in *C. albicans* and *A. nidulans.* The HSAF can disrupt polarized growth and induce cell wall thickening of *A. nidulans* that negatively affects hyphal growth [13]. After being pretreated by HSAF, ROS (reactive oxygen species) was accumulated in *C. albicans* resulting in cell apoptosis and cell death [14]. Our group found that its fatal concentration against various pathogens was different, but most of them are similar to chemical fungicide. We have increased the yield of HSAF to 500 mg/L through improving the fermentation conditions. These bacteria products containing HSAF were well applied in several pear orchards to control Valsa canker disease, pear black spot disease, pear scab and black rot of pear, and its effect was similar to other chemical fungicides. Therefore, *L. enzymogenes* and HSAF have great potential to be used as biological control in the management of plant diseases.

Recently, several key components of HSAF biosynthesis pathway were identified; HSAF production was increased by optimizing the culture conditions and genetically modifying the production strains [15,16,17,18,19]. However, mechanisms of how this antifungal compound affects the cellular metabolisms of fungi upon treatment are not well understood. Several reports suggested that HSAF affect ROS-mediated apoptosis [14], nutrient acquisition, hyphal tip elongation [13]; however, detailed effect in gene expression at a global level has not been characterized.

*Alternaria alternata* (Fries) Keissler is a vital phytopathogen that causes black spot disease of several crops such as apple [20], tobacco [21], strawberry [22] and pear [23]. Similar to *L. enzymogenes* strains, it is ubiquitously present in soil, air, water and on plant surfaces such as leaves, rots, and trunks [24]. Recently, black spot disease and leaf defoliation caused by the pear pathotypes of *A. alternata* are prevalent in orchards of southern China, resulting in a mass loss in pear production. Previously, our group determined that the *L. enzymogenes* strain OH11 and its metabolic product HSAF can inhibit mycelial growth and spore germination of *Valsa pyri*; and the antifungal agents produced by this strain can successfully reduce the disease incidence of black spot disease on pear leaves. However, the antifungal mechanism of HSAF has not been clear until now, especially against *A. alternata*.

To further elucidate the antifungal mechanisms of HSAF against *A. alternata*, in this study, we performed transcriptomic analysis of *A. alternata* strain HN-5 in the presence and absence of HSAF. The elucidation of the antifungal mechanism of HSAF not only improved our understanding of the interaction of two plant associated microbes (*A. alternata* and *L. enzymogenes*), but also provide the knowledge support of future development of the HSAF as an antifungal biocontrol agent.

## 2. Results

### 2.1. Collection of the Black Spot Disease Pathogen A. alternata Strains from Infected Pear Leaves in Southern China

A total of 150 pear leave samples with typical black spot disease symptoms were collected from pear orchards in 11 provinces of China between 2014 and 2017. A total of 125 *A. alternata* strains were isolated from these samples and were characterized by Internal Transcribed Spacer (ITS)-based phylogeny. The pathogenicity of these strains was compared on pear leaves. HN-5 strain, which displays strong virulence phenotype on pear, was further characterized for its sensitivity upon HSAF treatment as describe below.

### 2.2. The Growth Inhibitory Effects of A. alternata by HSAF

**Figure 1:**
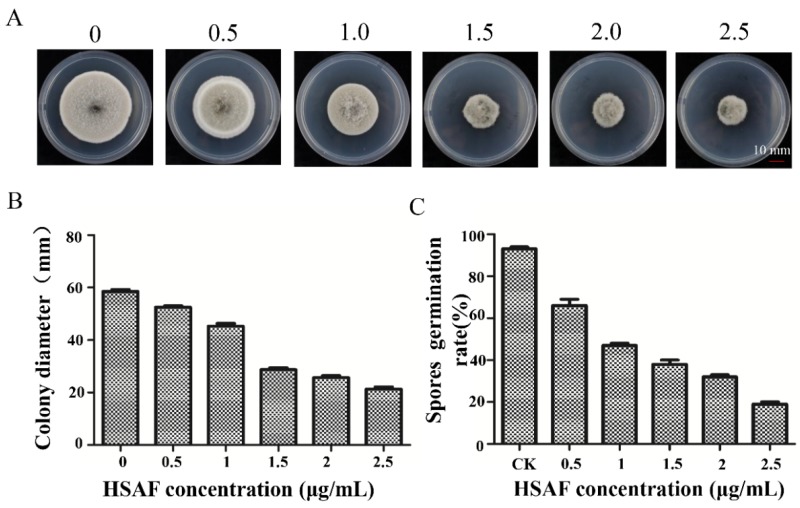
Inhibitory effect of HSAF (Heat-stable activity factor) on *A. alternata*. (**A**) The inhibitory effect of HSAF on mycelia growth of HN-5. HSAF was extracted and purified by HPLC (High Performance Liquid Chromatography) using a linear gradient program (Y = 4 × 10^−5^ X + 32.385, X indicates mAu, Y means concentration). The HN-5 strains were grown on PDA plates supplemented with HSAF (0, 0.5, 1.0, 1.5, 2.0, 2.5 μg/mL). The colony morphology was documented by a camera after cultured for 5 days. (**B**) Inhibitory effect of HSAF on mycelia growth of HN-5. The colony sizes were measured using rulers with cross method, and the data of the colonies’ sizes were analyzed using formats in Microsoft Excel 2010. (**C**) Inhibitory activity of HSAF to germination of HN-5. Spores were collected after being cultured on PDA for 8 days, collected and divided into the tubes with 1 mL PDB medium containing HSAF (0, 0.5, 1.0, 2.0, 2.5 μg/mL), the germinated spores were counted using hemocytometer, and the data were also analyzed using formats in Microsoft Excel 2010 (Microsoft, Redmond, Washington, DC, USA). Bar = 10 mm. Each experiment was repeated at least three times.

To evaluate whether HSAF exert any growth inhibition of HN-5, HN-5 was cultured on PDA (Potato Dextrose Agar) amended with HSAF at different concentrations (0, 0.5, 1.0, 1.5, 2.0, 2.5 μg/mL). Colony sizes and morphology were documented at 5 days post inoculation. We observed that the sizes of the colonies of HN-5 were reduced with increased concentration of HSAF (Figure 1A,B). Furthermore, we determined effect of HSAF to the germination of HN-5 spores. Similar to the effect of HSAF on the vegetative growth of HN-5, the spore germination was also reduced with increased concentrations of HSAF (Figure 1C). These results demonstrate that HSAF inhibit both the growth and germination of *A. alternata* and may act as good biofungicide against this fungus.

### 2.3. Cellular Modification of A. alternata against HSAF

**Figure 2:**
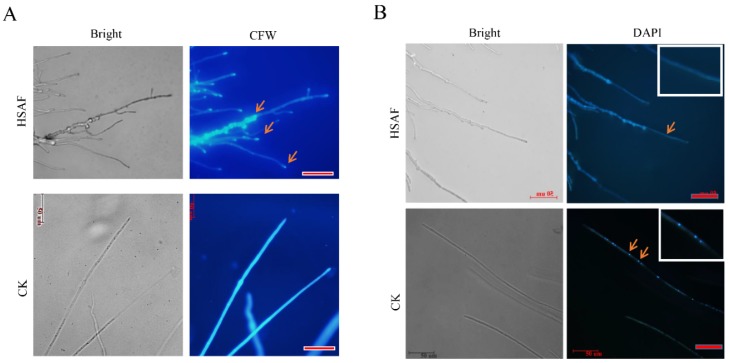
HSAF induced cell wall thickened and caused cell apoptosis in *A. alternata.* (**A**) Mycelial microscopic morphological characteristics and cell wall staining by CFW (Calcofluor white). The strains were cultured on coverslips with a layer PDA medium amended with HSAF or methanol (CK) at 25 °C for 24 h, then staining by CFW. The pictures were taken by fluorescent inverted/phase contrast microscope (ZEISS). The arrows indicate the well-staining areas that were similar to the control. Bar = 50 μm. (**B**) Hyphal apoptosis was induced by HSAF. The strains were cultured on the conditions same to the ways above. The mycelia were staining by DAPI. The pictures were also taken by fluorescent inverted/phase contrast microscope (ZEISS). The small panels were enlarged area from these arrows indicated sites. Bar = 50 μm.

Previous studies showed that HSAF inhibits hyphal tip elongation of *A. nidulans* and *Candida albicans* [13] and induces ROS-mediated apoptosis. To confirm whether HSAF has same effect to *A. alternata*, we first compared the mycelial morphology with (HSAF treatment group, abbreviated HSAF in full text) and without HSAF treatment (adding methanol as the control check, abbreviated CK in full text) using microscopy. Similar to previous observations, thickened mycelia and increased branching of hyphal tips were observed in the HSAF-treated sample (Figure 2A). When stained by Calcofluor white (CFW), the mycelia and hyphal tips of the HSAF-treated *A. alternata* were clearly bright and were similar to the non-HSAF-treated mycelia. However, the hyphal branches cannot be stained well. The staining results indicate that HSAF may promote the fungal cell wall biosynthesis but inhibits hyphal elongation (Figure 2A). Additionally, nuclear degradation is a key step for cell apoptosis. The DAPI (4′,6-diamidino-2-phenylindole) staining demonstrated that the nucleus of hypha vanished in the HSAF-treated samples (Figure 2B), indicating that HSAF also induced cell apoptosis in *A. alternata*. These data imply that HSAF induced cell apoptosis and remodified the mycelial morphology.

### 2.4. RNA Sequencing and Novel Genes Prediction

We prepared mycelia that treated with HSAF or methanol, three replicates of which were constructed. RNA sequencing produced a total of 161.80 million 150-bp paired-end reads. After removing adapter and filtering out low-quality reads, 22.87 Gb of clean data was acquired. The Q20 value was more than 94.59%. The latest genome of *A. alternata* was used as the reference [25]. The GC content of each sample was higher than that of the reference strain. Reference-guided transcriptomic assembly was performed based on the high-quality filtered reads. Clean reads were mapped to this genome with the ratio ranging from 82.42% and 83.93% (Table S1). Approximately 82.19–83.61% of the total mapped reads were unique alignments. Multi-aligned reads were removed and only the unique reads were used for further analysis. Since the percentage of unmapped reads was high, we analyzed these reads and determined 146 new transcripts that encode proteins with up to 100 amino acids. Of these transcripts, 98 are encoding genes, but only 27 have Nr hits against the Nr database (Figure S1). These results indicate the good quality of the RNA sequencing of *A. alternaria*.

### 2.5. Differentially Expressed Genes Analysis and Function Annotation

**Figure 3:**
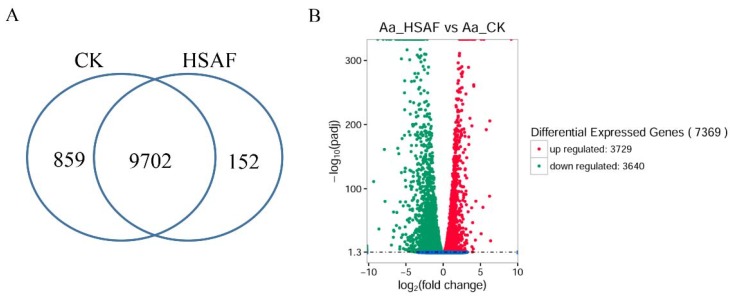
Gene characterization and analysis for differential expressed genes. (**A**) Venn dialog of expressed genes from genome analysis. Venn diagrams depict the numbers of expressed genes in *A. alternata* strains that were treated by HSAF or adding methanol as a control (CK). The numbers indicate the counts of common or unique expressed genes. (**B**) Volcano Plot of DEGs (differentially expressed genes). Red spots mean up-regulated genes; green indicates down-regulated genes. Green spots indicate down-DEGs, Red spots indicate up-DEGs. *X* axis represented as fold change of DEGs, and *Y* axis revealed the adjust *p*-value (*p*-adj).

To explore the genes in response to HSAF, we compared the gene expression before and after HSAF treatment at a global level. The Venn diagrams shows 10,713 expressed genes that are unique or common in the HSAF group or the control group (Figure 3A). For each treatment, three biological replicates were included, and the Pearson correlation between the samples was calculated. R^2^ value up to 0.991 implied the reliability of the analysis (Figure S2). A total of 7369 differentially expressed genes (DEGs) were identified with significant expression differences between the HSAF-treated and control groups (*p*-adj < 0.05) (Table S2). Among them, 3729 were up-regulated and 3640 were down-regulated upon HSAF treatment (Figure 3B).

### 2.6. Gene Ontology (GO) and KEGG Pathway Enrichment of DEGs

**Figure 4:**
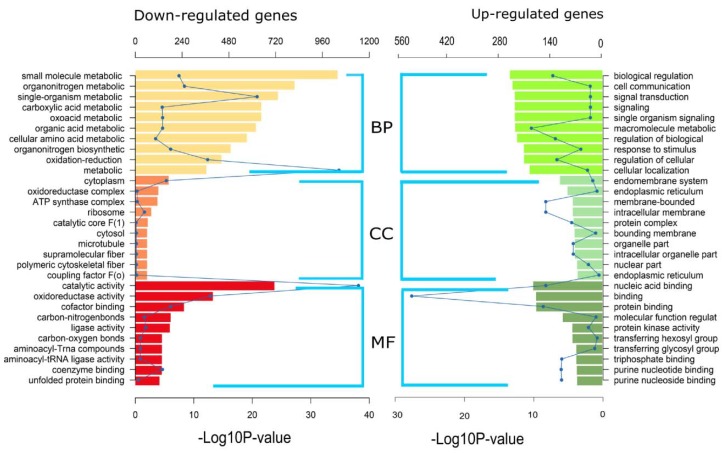
GO analyses of DEGs. The top 10 enriched GO terms of each category are shown on the graph. The curve means number of DEGs, and the column indicates *Q*-value. BP: biological process, CC: Cellular component, MF: molecular function. Upon *X* axis indicates DEGs numbers representing as curve, the below *X* axis means −log10P-value representing as column. The left or right *Y* axis indicates GO category.

GO enrichment analysis was performed to identify DEG sets in response to HSAF based on GO category involved in biological process (BP), cellular component (CC) and molecular function (MF). The down-regulated DEGs with GO terms including biological process and molecular function were clearly more than the up-regulated DEGs (Figure 4, Table S3). The top 10 enriched GO terms of each category are determined (Figure 4). Metabolic process, catalytic activity and membrane related terms were the most abundant in the three categories BP, MF and CC, respectively.

**Figure 5:**
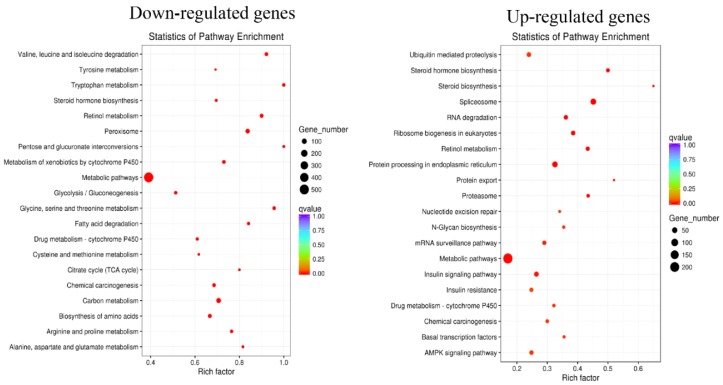
KEGG pathway enrichments of DEGs. The left panel shows 20 enriched KEGG terms for down-regulated DEGs responding to HSAF. The right panel presents up-regulated DEGs. The legend is shown on the picture.

To confirm the pathway disrupted by HSAF, we performed KEGG analysis, which classified the DEGs into 269 KEGG pathways, with the most enriched pathways presented in Figure 5. Of the down-regulated DEGs, genes involved in amino acid metabolism, drug metabolism, citrate acid cycle (TCA cycle) and carbon metabolism were enriched, the function of which are related to growth, energy, and drug degradation of *A. alternata* (Figure 5, Table S4). Pathways with the functions related to ubiquitin-mediated proteolysis, RNA degradation, protein processing in endoplasmic reticulum, Cytochrome P450 and AMPK signaling were enriched in the up-regulated DEGs (Figure 5).

### 2.7. Genes Involved in Central Carbon Metabolism Responded to HSAF

**Figure 6:**
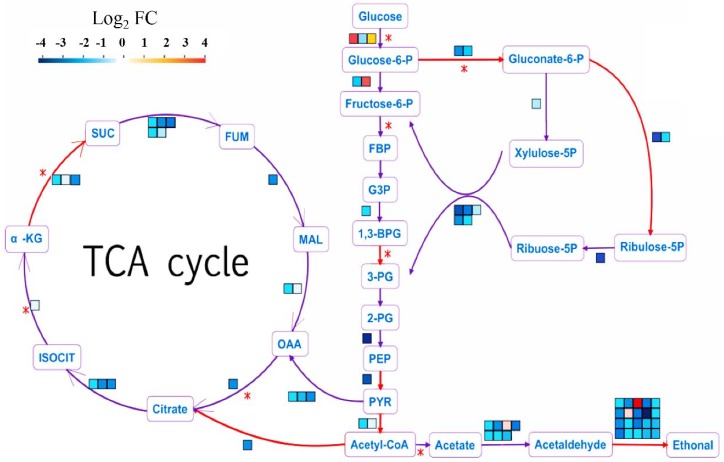
Glycolysis pathway and TCA cycle in response to HSAF. The red asterisk indicates key pacemaker enzyme for the pathways. The red arrow represents the exergonic reaction. One square denotes one gene in related reaction and its color implies the gene’s expression level. The asterisks (*) indicate the key reaction. FC (Fold change of RPKM (HSAF)/RPKM (CK)), PGI1 (glucose-6-phosphate isomerase), FBA1 (fructose-bisphosphate aldolase), FBP1 (fructose-1,6-bisphosphatase), TDH1 (glyceraldehyde-3-phosphate dehydrogenase), PGK1 (phosphoglycerate kinase), ENO1 (enolase), PYK1(pyruvate kinase), PDA1 (pyruvate dehydrogenase E1 component alpha subunit), PDB1 (pyruvate dehydrogenase E1 component beta subunit), PDC (pyruvate decarboxylase), CIT1 (citrate synthase), ACO1 (aconitate hydratase), IDH1 (isocitrate dehydrogenase), IDH2 (isocitrate dehydrogenase), KGD1 (2-oxoglutarate dehydrogenase), SDH1 (succinate dehydrogenase), SDH2 (succinate dehydrogenase), FUM1 (fumarate hydratase), MDH1 (malate dehydrogenase). For the metabolites (red fonts), these abbreviations represent: glucose-6-P (glucose 6-phosphate), gluconate-6-P (gluconate-6-phosphate), fructose-6-P (fructose 6-phosphate), FBP (fructose 1,6-bisphosphate), G3P (glyceraldehyde 3-phosphate), 1,3 BPG (glycerate 1,3-diphosphate), PG (phosphoglycerate), PEP (phosphoenolpyruvate), PYR (pyruvate), Acetyl-CoA (acetyl coenzyme A), CIT (citrate), ISOCIT (iso-citrate), α-KG (alpha-ketoglutarate), SUC (succinate), FUM (fumarate), MAL (malate), OAA (oxaloacetate).

Since the KEGG analysis showed that the terms including central carbon metabolism and TCA cycle were the most redundant in down-regulated DEGs, we further analyzed the impact of HSAF on these pathways. The number and expression levels of genes involved in crabtree-effect-related metabolic enzyme were analyzed. In *A. alternata*, we observed that 68 DEGs were related to carbon metabolism (Table S5), and 92.6% genes were down-regulated. Specifically, proteins catalyzing key chemical reactions such as α-KG (α-ketoglutarate) to succinate, acetyl coenzyme A to citrate, glycerate 1, 3-diphosphate to phosphoglycerate, glucose-6-phosphate to gluconate-6-phosphate that producing energy were important for cell surviving and mycelia growth (Figure 6). 18 genes involved in anaerobic respiration (the reaction of acetaldehyde to ethanol) were also down-regulated, indicating that the anaerobic respiration was also blocked by HSAF (Figure 6). However, the glucose transporter involved in the reaction of glucose to glucose-6-P, was up-regulated, implying that the glucose can transfer to the cell from outside (Figure 6). These results demonstrated that HSAF might block the glycolysis pathway and TCA cycle, causing limited energy for hyphal growth and metabolism.

### 2.8. Chitin Synthases Encoding Genes Were Predominately Up-Regulated upon HSAF Treatment

**Figure 7:**
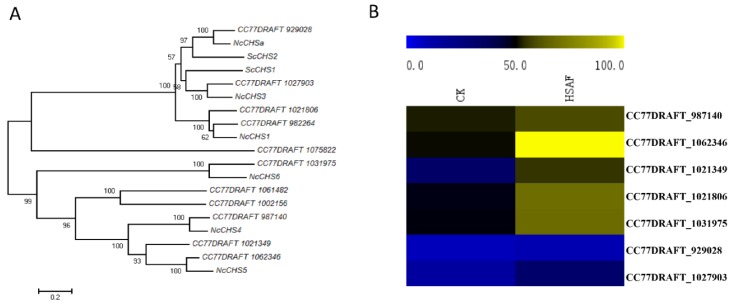
The chitin synthases characterization and their expression levels. (**A**) Phylogenetic analysis of fungal CHSs. Protein sequences of ScCHS1 (SCRT_02929) and ScCHS2 (SCRT_03322) were downloaded from *Saccharomyces cerevisiae* genome database. Protein sequences of NcCHSa (EAA32767.3), NcCHS1 (EAA32102.2), NcCHS3 (EAA32006.2), NcCHS4 (ESA44002.1), NcCHS5 (EAA27097.2), and NcCHS6 (EAA32522.3) were downloaded from the NCBI database. The sequences were aligned using the ClustalW tool [26] and the tree was constructed by the neighbor-joining method using MEGA 5.0 software [27]. Confidence levels above the nodes were obtained from a 1000 bootstrap analysis. (**B**) Expression levels of *CHS* genes. The expression data of *CHS* genes were obtained from the DEGs analysis. The heat map was constructed by MeV 4.8 software (MultiExperiment Viewer, Boston, MA, USA) with the original FPKM values of each gene.

Chitin serves as the major component of the cell wall in many fungi (Classification of fungal chitin synthases). Chitin synthases (CHSs) are enzymes that catalyzed the reagents of chitin synthesis. Staining results showed mycelia were thickened. Most of the genes involving in glycolysis pathway and TCA cycle were down-regulated. These results may imply that glucose may use for thickening of the cell wall. To validate the hypothesis, we try to analyze the expression levels of CHSs family in *A. alternata*. However, the CHSs have not identified and classified well in *A. alternata* besides CHS2 and CHS6. According to the proteins that well-classified in *Saccharomyces cerevisiae* and *Neurospora crassa*, we obtained 11 chitin synthase and characterized them by constructing phylogeny tree using Neighbor-joining method (Figure 7A, Table S6). Later we acquired the FPKM (fragment per Kilobase of exon model per Million mapped Fragment) value from the DEGs analysis and a heat map of the gene expression levels was drawn, that showed all the *AaCHS* expression levels were up-regulated responding to HSAF except four genes (CC77DRAFT_1061482, CC77DRAFT_1002156, CC77DRAFT_1075822 and CC77DRAFT_982264) with low FPKM values were considered unexpressed genes (Figure 7B). These results accord with the phenotype of mycelia under HSAF treatment and further illustrate that the cell wall was enhanced in response to HSAF.

### 2.9. Nitrogen-Metabolism-Related Genes Were Predominately Down-Regulated upon HSAF Treatment

**Figure 8:**
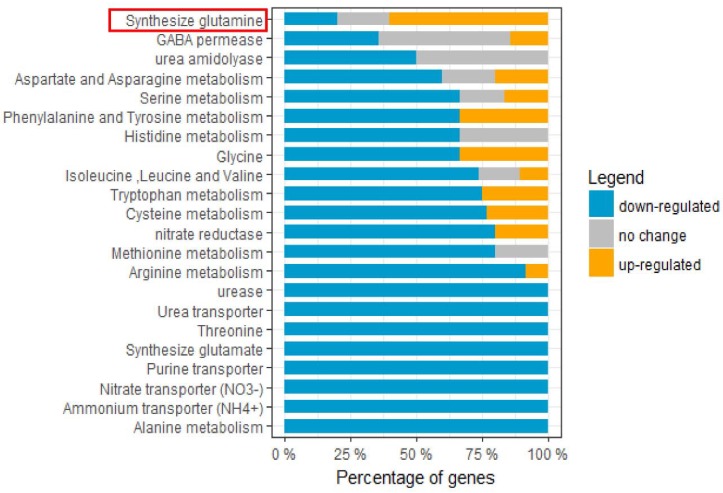
Nitrogen metabolism pathway responding to HSAF. The *X* axis indicates percentage of DEGs involving nitrogen metabolism. The *Y* shows the terms of GO category. The red box emphasizes that only one term, in which the number of up-regulated genes encoding synthesize glutamine were more than the number of down-regulated genes.

Due to the enrichment of amino acid metabolism response to HSAF, we further analyzed the DEGs involved in nitrogen metabolism. The nitrogen-metabolism-related proteins were predicted based on the database of *U. maydis* [28] and were classified into 22 terms including mineral nitrogen metabolism and amino acid metabolism. The percentage of up- and down-regulated genes was calculated (Figure 8, Table S7). Most genes exhibited down-regulated response to HSAF, except genes encoding “Synthesize glutamine” proteins (Figure 8). Specifically, all proteins involved in mineral nitrogen metabolism including urease, urea transporter, nitrate transporter and ammonium transporter were down-regulated (Figure 8). These results suggest that HSAF may disrupt the nitrogen metabolism of *A. alternata*.

### 2.10. Signaling Pathway Responding to HSAF

**Figure 9:**
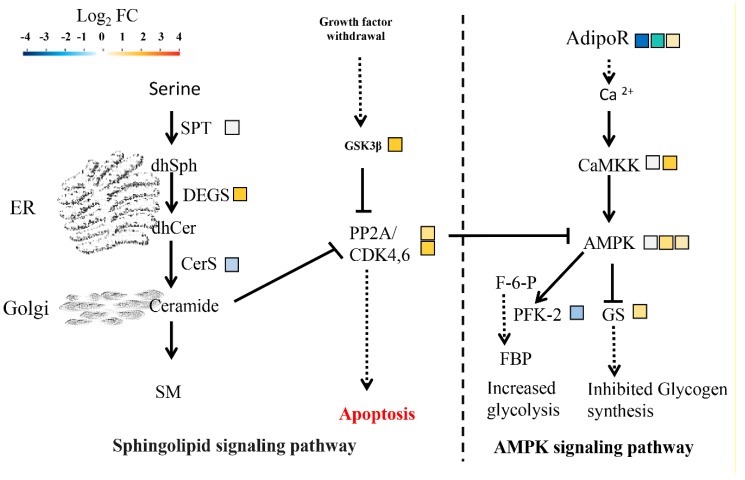
Signaling pathways were regulated by HSAF. The heat graphs indicate fold change of related genes according to the ratio with Log_2_ FC. One square denotes one gene in related reaction and its color implies the gene’s expression level. The dotted arrows indicate uncertain pathways. The solid arrows indicate the known pathways. FC (Fold change of RPKM (HSAF)/RPKM (CK)), Sphingolipid signaling pathway: *SPT*: serine palmitoyltransferase, *CerS*: ceramide synthase, *DEGS*: sphingolipid 4-desaturase/C4-monooxygenase, *PP2A*: protein phosphatase 2A, *GSK3β*: glycogen synthase kinase 3 beta, *BCL-2*: apoptosis regulator. AMPK signaling pathway: *CaMKK*: calcium/calmodulin dependent protein kinase 2, *AMPK*: AMP-activated protein kinase, *GS*: glycogen synthase, *AdipoR*: adiponectin receptor, *PFK-2*: fructose-2, 6-biphosphatase 2.

Previously, the proteins BarA, a ceramide synthase, involved in the sphingolipid signaling pathway were considered as a target of HASF [13,29]. Sphingolipid is the product of serine. To confirm whether the sphingolipid signaling pathway of *A. alternata* responded to HSAF, we analyzed the expression of genes participating in the sphingolipid signaling pathway and sphingolipid metabolism. The pathway was predicted according to the KEGG pathway that was utilized previously. Most genes were down-regulated; especially the ceramide synthase, but sphingolipid 4-desaturase and two protein phosphatase 2A (PP2A)-encoding genes were significantly up-regulated (Figure 9, Table S8). PP2A is a key hub for several signaling pathways including the AMPK signaling pathway and the cell cycle pathway. Glycogen synthase kinase 3 beta (GSK3β) inhibited PP2A activity. PP2A could inhibit AMPK phosphorylation (Figure 9). The AMPK signaling pathway is important in regulation of cellular metabolism such as glycogen synthesized and glycolysis. Therefore, we also analyzed the transcript levels of genes involved in AMPK signaling pathway. We observed that these genes were up-regulated under HSAF treatment besides fructose-2, 6-biphosphatase 2 and two adiponectin receptor-encoding genes (Figure 9). In summary, multiple signaling pathways of *A. alternata* were disrupted by HSAF.

### 2.11. RT-qPCR Validation of DEGs

**Figure 10:**
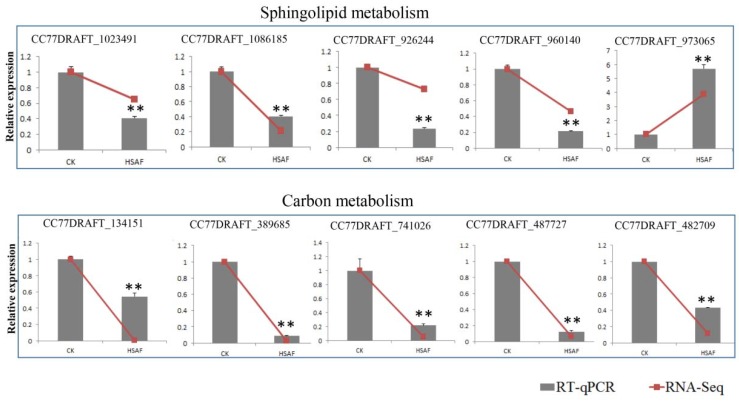
RT-qPCR validations for genes participating in sphingolipid and nitrogen metabolism. The ten genes were using for RT-qPCR analyses. Columns indicate the relative expressions of genes were tested by the RT-qPCR, and linear graphs show the mean relative expressions data of genes that were predicted by RNA-seq. The graph showed 5 genes’ expression levels under HSAF treatment, and 5 genes’ relative expression is shown on the graph below. Relative expression of each gene was analyzed by RT-qPCR, normalized to *actin* expression levels and the relative expression ratio was calculated as the fold change (2^−ΔΔ*C*t^) compared to the wild type (**, *p* < 0.01, *t*-test). The relative expression levels of genes in RNA-seq were calculated using “CK: the mean RPKM (CK) value/the mean RPKM (CK) value or HSAF: the mean RPKM (HSAF) value/the mean RPKM (CK) value” The mean relative expression levels of three replicates from RT-qPCR analyses were used for drawing graph. Each experiment was repeated at least three times.

To confirm the DEGs identified in the transcriptomic analysis, the expression of ten DEGs with functions in sphingolipid metabolism and sugar transporter were determined using RT-qPCR. In agreement with the transcriptomic analysis (Table S9), 9 of the 10 genes also showed significant down-regulation in HSAF-treated samples, except for CC77DRAFT_973065 (XP_018380930.1) (Figure 10, Table S10). Furthermore, their expression levels are consistent with the transcriptome analysis (using the mean relative expressions data of genes) with a high Pearson correlation (R = 0.97) (Table S10). These data further demonstrate that HSAF might block sphingolipid metabolism and carbon metabolism.

## 3. Discussions

*A. alternata* is an economically important pathogen to the sand pear in China. We isolated HN-5 strain from diseased pear leaves and identified it based on rDNA-ITS (Internal Transcribed Spacer) analysis. This strain had a strong virulence to Cuiguan pear. Similar to other filamentous fungi, its growth can be inhibited by HSAF (Figure 1 and Figure 2). Because the pear leaves black spots disease widely occurs in the south of China and results in severe loss of yield and quality, development of effective management program of this disease is becoming an urgent priority. Chemical fungicides exhibit high phyto-toxicity and the residues of which also causes concerns for human health. HSAF is a natural antifungal agent, and chemosynthesis of HSAF has yet been achieved. Even though HSAF can be biosynthesized, its yield was very low which limits the large-scale production and its application in the field. Therefore, understanding its toxicology to fungal pathogens will benefit us in finding its targets, and improve its antifungal activity by adding an effective adjuvant. Previously, the mechanisms of antifungal activities of HSAF were analyzed in *M. oryzae, C. albicans* and *A. nidulans* [12,14]. For example, *L. enzymogenes* can invade the hyphae and conidia of *M. oryzae* that induced fungal cell death and changes of gene profiling in *M. oryzae* [12,14]. In *C. albicans* SC5314, β-tubulin maybe a target of HSAF, and HSAF induced the apoptosis through ROS accumulation [14]. The polarized growth of *A. nidulans* was disrupted by HSAF that led to loss of formin localization and membrane organization at hyphal tips. Two ceramide synthases mediated the effects of HSAF and regulates hyphal morphogenesis [29]. However, there are still a lot of questions to be answered. For example, different fungi have the same mechanism or various mechanisms in response to HSAF, so are there any other targets binding to HSAF? If the target is the β-tubulin, how does it mediate apoptosis and induced cell death? Here, we performed transcriptomic analysis of *A. alternata* to demonstrate the metabolism and signaling pathways that respond to HSAF, which provided some novel mechanisms of HSAF antifungal activities. Similar to other fungi [12,14], the hyphal growth of *A. alternata* was inhibited by HSAF, and the cell wall thickening and cell apoptosis were also induced in HSAF-treated mycelia. RNA sequencing was performed. According to transcriptomic analysis, we found that genes involving in carbon metabolism and TCA cycle, sphingolipid metabolism and nitrogen metabolism were dominantly down-regulated responding to HSAF toxicity; signaling pathways including AMPK pathway, cell cycle and sphingolipid signaling pathway involving apoptosis were disrupted by HSAF; cell wall synthase genes were up-regulated resulting in the thickening of cell wall against HSAF toxicity. We propose that a network including several new signaling pathways were demonstrated; they provided a new mechanism of HSAF toxicity, which will facilitate the understandings of the antifungal activities of HSAF against filamentous fungi.

Transcriptomic analyses have been used as an effective approach in understanding gene expression in fungi [30,31,32]. To understand the cellular response of *A. alternata* to HSAF, transcriptomic analysis was also used in this study. Transcriptomic analysis of growth inhibition mediated by *L. enzymogenes* strains or HSAF has been previously studied in *M. oryzae* and *C. albicans* [12,14]. However, a few fungal DEGs involving apoptosis and ROS accumulation were identified in the interaction between *L. enzymogenes* and fungi [12,14]. We observed that *A. alternata* strain HN-5 can grow on the medium with high concentration (10 mg/L) of HSAF, which indicates that *A. alternata* may have novel specific mechanisms to counteract HSAF toxicity that varied to other fungi. Furthermore, *A. alternata* strain HN-5 is a Chinese pear pathotype, which may differ from the *A. alternata* strains of pear pathotype or other *A. alternata* strains have been sequenced [33]. Therefore, the transcriptomic analysis of *A. alternata* is necessary, and it will enrich the knowledge regarding this pathogen. The latest sequence assemblies of *A. alternata* strains were concentrated on strain Z7 and strain SRC1lrK2f. The assembly of the tangerine pathotype of *A. alternata* strain Z7 reported 12,062 genes, 11,611 of them were orthologous genes to other sequenced species [33]. In the strain SRC1lrK2f, 13,466 protein sequences were reported [25]. Due to the predicted protein in the strain SRC1lrK2f is more than strain Z7, we selected the assembly and annotated proteins from this strain for transcriptomic analysis in this study. In this transcriptome analysis, the ratios of mapped reads ranged between 82.42% and 83.93%, which implies considerable phylogenetic distance between the HN-5 strain and reference strain SRC1lrK2f (Table S1). We identified 10,713 expressed genes, less than 13,466 genes characterized in the reference genomics [25]; however, some of them were novel genes (Figure S1) that was not predicted in reference genome of *A. alternata* SRC1lrK2f. Because of the use of the correct *p*-value (*p*-adj < 0.05) for screening DEGs, the number of DEGs was up to 68.79%. Most GO enrichments were similar to the transcriptomic analysis of *C. albicans*; for example, DEGs in the terms of oxidoreductase activity, macromolecule metabolism and catalytic activity were abundant in both *A. alternata* and *C. albicans* responding to HSAF (Figure 4) [14]. However, no KEGG pathways were predicted in *C. albicans*. The KEGG pathway enrichments for *A. alternata* were similar to the plant-derived antifungal compound honokiol for the fission yeast [30]. The membrane-related terms and metabolic process were enriched in *A. alternata*, which demonstrates that different fungi have unique responses to the HSAF toxicity. These analyses imply that HSAF has its distinct roles to inhibit the fungal growth.

Fungal cell wall is important for the morphogenesis and viability of the fungal cell [34,35,36,37]. They mainly consist of chitin, sporopollenin, melanins, aminopolysaccharides, β-1, 3-glucan, and mannoproteins [35]. Chitin is the product of CHSs in fungi. The expression levels of *CHSs* reflect the accumulation of Chitin. Cell wall thickening induced by HSAF was observed in several fungi, such as *Bipolaris sorokiniana*, *A. nidulans* and *Cryptococcus neoformans* [13]. Similar to the other fungi, the cell wall thickening was also observed on HSAF-treated mycelia and hyphal tips of *A. alternata,* but not hyphae. Accordingly, the expression levels of *CHSs* were also up-regulated under HSAF treatment. These results further determined that fungal cell wall thickening was an essential and a conserved response to HSAF treatment in fungi. The cell wall thickening mechanisms were discussed previously. It was considered that cell wall thickening may require a putative HSAF sensor that activates a signal pathway(s) which induces cell wall synthesis. Cell wall thickening may be mediated by signaling pathway and regulated by several transcription factors [38,39,40,41,42]. In *A. nidulans*, the experiments validated that thickened cell wall patches could not be re-utilized rapidly for fungal growth on non-HSAF-treated medium, but the hyphal tips can [13]. This result correlates with our data: the cell wall of hyphae but not hyphal tips cannot be stained well by Calcofluor (Figure 2A). Cell wall thickening may lead to consumption of energy and materials which blocks nutrient metabolism for fungal growth, function as a negative regulator of cell growth [13]. In this study, we found that genes involving carbon metabolism and nitrogen metabolism pathways were dominantly down-regulated under HSAF treatment, which further demonstrates that cell wall thickening blocks energy and nutrient utilization of new hyphae. These results further confirmed that HSAF function as an inhibited factor for fungal growth, but an inducer of cell wall thickening.

In fungi, TCA (tricarboxylic acid) cycle ligase the amino acids or ammonium metabolism and glycolysis, which are sensed and taken up by several signaling pathways including protein kinase A and AMPK [43]. TCA cycle is a key pathway that responding the toxicity of fungicides such as antifungal compound honokiol and cinnamon oil [30,43]. DEGs involving in TCA cycle were dramatically down-regulated (Table S5). In particular, the proteins catalyzing key chemical reactions producing energy were important for cell survival and mycelia growth were also down-regulated (Figure 6 and Figure 8). These results further validated that HSAF can inhibit fungal growth through blocking the central carbon metabolism and energy liberation. TCA cycle and carbon metabolism were disrupted by HSAF, which also resulted in inhibition of nitrogen metabolism that is downstream of TCA cycle, implying that HSAF functions as a negative regulator of main metabolism in fungi, which provide novel insights to the mechanism of HSAF antifungal activities.

Sphingolipid metabolism and the sphingolipid signaling pathway are well known for regulating apoptosis and autophagy [44,45,46]. BarA, a ceramide synthase, mediated cell wall thickening that induced by HSAF [13]. However, it is still not clear that BarA or sphingolipid directly or indirectly binds with HSAF. In this study, most genes involved in sphingolipid metabolism or sphingolipid signaling pathway were down-regulated, besides DEGS (Figure 9 and Figure 10A). Serine is the resource for sphingolipid synthesis, which is catalyzed by serine palmitoyltransferase [47,48]. The expression level of the gene encoding ceramide synthase which was considered as a target was down-regulated under HSAF treatment, but sphingolipid 4-desaturase/C4-monooxygenase encoding gene which is upstream of CerS was up-regulated induced by HSAF. These results mean that the sphingolipid signaling pathway was disrupted by HSAF and ceramide accumulation was reduced. However, the previously studies descripted that sphingolipid or ceramide were involving in apoptosis and cell wall thickening. Ceramide inhibit the activity of PP2A which is vital for apoptosis and cell cycle. We deduce that CerS may cause the reduction of ceramide, which was sensed by PP2A and then induce apoptosis. These results demonstrated that the apoptosis of *A. alternata* caused by HSAF may be a result of HSAF’s disruption of the sphingolipid signaling pathway (Figure 2B).

Interestingly, the DEGs involved in the AMPK signaling pathway, which positively regulates fungal glycogen synthesis [49], were up-regulated (Figure 9). This finding is in keeping with the observations that the cell wall of the mycelia or hyphae was thickening, responding to HSAF (Figure 2A). PP2A senses signals from growth withdrawal factor, and is a hub for the AMPK signaling pathway, cell cycle and sphingolipid signaling pathway [50,51,52]. Multiple pathways were disturbed by HSAF. However, how fungal cells perceive the signal of HSAF and modulate these cellular pathways in fungal cells is not clear. Adiponectin receptor protein 1 (AdipoR1), is a receptor that localized on the membrane and is well-known to regulate multiple biological progresses or metabolic pathways as an element AdipoR-AMPK pathway in mammals [53,54,55,56]. AdipoR1 recognizes deleterious factors, such as high glucose, salt, hormone and other nutrient stress [53,55,57], and modulate downstream responses such as cell growth and apoptosis [54,55,58]. In our study, the expression level of an *AdipoR* was nine times lower in the HSAF treatment than CK (Figure 9). We hypothesize that AdipoR may serve as an important receptor in HSAF sensing and subsequently modulation of the downstream responses.

## 4. Materials and Methods

### 4.1. HSAF Extraction and Quantification in L. enzymogenes OH11 Cultures

HSAF was extracted and quantified in *L. enzymogenes* OH11 using a previously described method with minor modifications [59]. The OH11 strains were cultured in 100 mL of 10% TSB at 28 °C in a rotary shaker at 220 rpm for 2.5 days. After centrifugation (10,000× *g* at 4 °C for 30 min), we collected the supernatant of the cultures. Three milliliter aliquots of fermentation samples of *L. enzymogenes* OH11 were withdrawn from the flasks and adjusted to pH 2.5 with HCl. Ethyl acetate was added to the acidified broth in a 1:1 ratio and was vortexed at 2000 rpm for 1 min. After centrifugation, 1 mL of the solvent layer containing HSAF was separated and ventilated to dryness in a fume hood. The HSAF crude extract was re-dissolved in 1 mL of methanol and used for High Performance Liquid Chromatography (HPLC) analysis using an InterSustainSwift C18 column (5 μm, 250 × 4.6 mm; Shimadzu, Japan) with detection at 318 nm. Pure water and acetonitrile containing 0.04% (*v*/*v*) TFA were used as the A and B mobile phases, respectively. A linear gradient program used a flow rate of 1.0 mL/min. Finally, the absorption peak areas were used to express the amount of HSAF in the extracts for comparisons with each other.

### 4.2. Measurement of Growth Inhibition of A. alternata by HSAF

The HN-5 strains were grown on PDA plates supplemented with HSAF (0, 0.5, 1.0, 1.5, 2.0, 2.5 μg/mL). After 5 days, the colony sizes were measured with a ruler using the cross method, and the colony morphology was documented by a camera. For measurement of spore germination, *A. alternata* spores were collected from mycelia cultured on PDA medium for more than one week. The collected spores were filtered using Miracloth and divided equally into five tubes containing 1 mL PDB with different concentrations of HSAF. After 24 h of incubation at 25 °C, the germinated spores were counted using a hemocytometer.

### 4.3. Staining of Mycelia Using Calcofluor White and 4′,6-Diamidino-2-phenylindole

The mycelia of *A. alternata* were cultured on the coverslips (Eagle) with a thin layer of PDA containing 0.5 mg/mL HSAF (HSAF) or equal volume of methanol (the control check, CK); after 24 h, the mycelia were stained using 1 mg/mL CFW (Calcofluor white) and 100 ng/mL DAPI. Images of the mycelia were obtained under an inverted microscope (ZEISS, Oberkochen, Germany) with a digital camera.

### 4.4. Sample Preparation for RNA Sequencing

The *A. alternata* strains were cultured on PDA (Potato Dextrose Agar) medium at 25 °C for 2 days. The fresh small fungal plugs were transferred to 100 mL PDB (Potato Dextrose Broth) and cultured for 48 h. Later the mycelia were collected and transferred to PDB adding 0.5 μg/L HSAF as treatment or adding methanol which was used for dissolving HSAF as a control, after 24 h, the mycelia were collected and stored at −70 °C. Three biological replicates of each group were performed. Total RNAs of each sample were extracted using the RNAsimple Total RNA kit (Tiangen, Nanjing, China), and their quality and concentration were determined by agarose gel electrophoresis and by NanoDrop, ND-1000 (Table S11, Figure S3). cDNA was synthesized using a PrimeScript reagent Kit (TaKaRa, Otsu, Shiga, Japan) according to the manufacturer’s instructions.

### 4.5. RT-qPCR Validation

RT-qPCR experiments were performed on an ABI Prism 7300 Fast Real-Time PCR System (Applied Biosystems, Carlsbad, CA, USA) using the SYBR Premix ExTaq reagent (TaKaRa). The primers used for the qPCR are presented in Table S12. The relative gene expression levels were calculated using comparative threshold cycle method (2^−ΔΔ*C*t^) and were normalized by *actin* [60]. Correlation between RNA-seq and RT-qPCR was calculated based on the fold changes of RT-qPCR and RNA-seq by format in Microsoft EXCEL2010 (Microsoft, Redmond, Washington, DC, USA).

### 4.6. cDNA Library Preparation and Sequencing

cDNA library preparation was generated according to the standard protocol of Illumina sample preparation method by the sequencing company (Newgenes, Shanghai, China). Briefly, the polies (A) mRNAs were purified using the Illumina TruSeq^®^ Stranded mRNA Sample Prep Kit (Illumina™, San Diego, CA, USA) and were enriched by oligo [61] beads using poly-T oligo-attached magnetic beads. Later they were fragmented to small pieces. The mRNA fragments were converted to first strand cDNA by reverse transcription using random primers, and then converted into double-stranded cDNA using DNA Polymerase I and RNAse H. After end repair, adapter ligation and agarose gel electrophoresis filtration, enrichment PCR was carried out on these DNA transcripts with adapter molecules on both ends. Finally, they were sequenced using Illumina HiSeq™ 2000/2500 platform (Illumina™, San Diego, CA, USA).

### 4.7. Bioinformatics Analysis

Raw data (raw reads) of FASTQ format were first processed through in-house Perl scripts. In this step, clean data (clean reads) were obtained by removing reads containing adapter, reads containing ploy-N and low-quality reads from raw data. Simultaneously, Q20, Q30 and GC content were calculated from the clean data. All downstream analyses were based on the clean data with high quality. Reference genome of *A. alternata* and annotation files of genes were downloaded from the NCBI website (Available online: https://www.ncbi.nlm.nih.gov/assembly/GCF_001642055.1). The index of the reference genome was built using Bowtie v2.0.6 [62] and pair-end clean reads were aligned to the reference genome using TopHat v2.0.9 [63].

### 4.8. Analysis for DEGs

The read number that mapped to each unigene was counted by HTSeq v0.6.1 [64]. The expression levels of the genes were calculated as FPKM based on gene length and read number [65]. For unigene DGE, the read numbers mapping to each unigene were counted by RSEM [66]. Differential expression analysis of HSAF treatment group and the control group (three biological replicates under each group) was performed using the DESeq R package (1.10.1) [67]. Genes with an adjusted *p*-value < 0.05 that was obtained using the Benjamini and Hochberg’s approach were considered as DEGs. The Pearson correlation between triple replicates of the control or HSAF treatments was calculated based on the RPKM value, and it showed high accordance because of R^2^ > 0.99 (Figure S2).

### 4.9. GO and KEGG Enrichment Analysis

GO (Gene Ontology) [68] analysis of DEGs was performed using the GOseq R package [68]. GO terms were assigned significantly enriched with corrected *p* value < 0.05. The first 30 GO enrichment terms of the DEGs were shown on graphs that were drawn based on the gene number and *Q*-value using R package. To enrich genes involved in the pathway, the KEGG pathways were predicted using the KOBAS software [69] with *p*-value < 0.05.

### 4.10. Prediction of Nitrogen-Metabolism-Related Genes in A. alternata

The nitrogen-metabolism-related genes in *A. alternata* HN-5 were predicted using Blastp [70] on the Broad Institute (Available online: http://www.broadinstitute.org). Known proteins from *Ustilago maydis* (Available online: mips.gsf.de/genre/proj/ustilago/) Genome Database and metabolic pathway catalog (Available online: http://www.genome.jp/kegg/) were used as queries. The orthologues HMMER was additionally performed according to searches for relevant Pfams [61], specifically PF13520 (Amino acid permease), PF01490 (Amino acid transporter), PF00909 (Ammonium Transporter Family), and PF00155 (Aminotransferase class I and II). After removing the redundant sequences, all obtained proteins were annotated against the NCBI and PFAM databases.

### 4.11. Prediction of Chitin Synthase Involving in Cell Wall Synthesis

Known chitin synthases from the *Saccharomyces cerevisiae* (Available online: www.yeastgenome.org) and *Neurospora crassa* Genome Database were used as queries. The available hidden Markov model (HMM) model of the Chitin synthase (CHS) domain was retrieved from the Pfam database (PF03142). The HMMs was used to screen the whole proteins of *A. alternata* with the *E*-value < 1 × 10^−5^. The predicted proteins were confirmed using BlastP against NCBI database. After removing the redundant sequences, all the CHSs of *A. alternata* were confirmed by a phylogenetic tree that was constructed by MEGA5.0 software using CHSs of *S. cerevisiae*, *N. crassa* as queries. The expression levels of CHS encoding genes were obtained from DEGs analysis. A heat map was generated by MeV software according to the RPKM value of CHS encoding genes.

## 5. Conclusions

**Figure 11:**
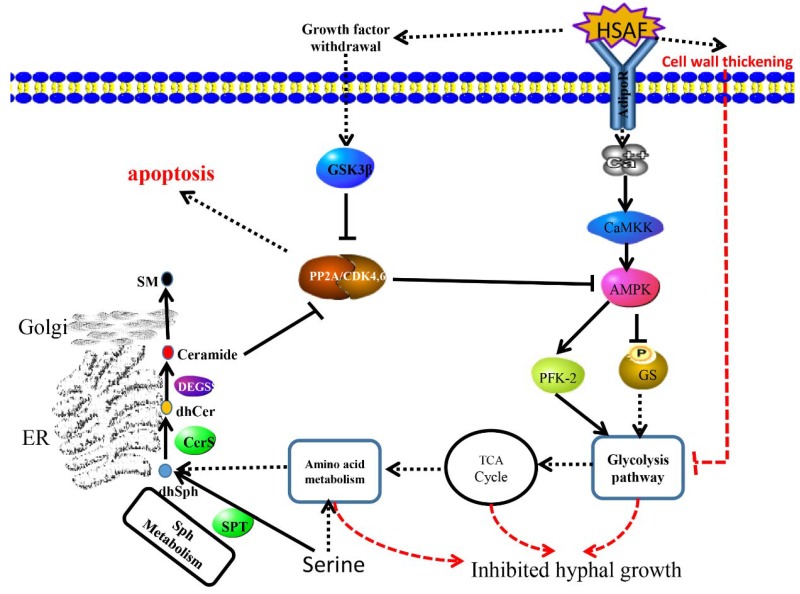
Hypothetical network responses to HSAF in *A. alternata*. The solid line represents the known reaction or step. The dotted line means uncertain steps in these pathways.

Taken together, our findings demonstrate that the natural antifungal agent HSAF can inhibit the hyphal growth of *A. alternata* through interfering with glycolysis, TCA cycle, nitrogen metabolism and sphingolipid metabolism, which may respond to the cell wall thickening that is resistant to HSAF treatment. These metabolisms block the new hyphal growth because of limited energy and materials. AdipoR may recognize HSAF and later induce the downstream signaling pathways (AMPK signaling pathway, sphingolipid signaling pathway and cell cycle) and metabolic pathways leading to cell wall thickening and apoptosis (Figure 11). These analyses demonstrate that a network was responding to HSAF treatment that provides novel global insights to HSAF antifungal activities.

## Supplementary Materials

Supplementary materials can be found at http://www.mdpi.com/1422-0067/19/7/1841/s1.

## References

[1] Sullivan R.F., Holtman M.A., Zylstra G.J., White J.F., Kobayashi D.Y. (2003). Taxonomic positioning of two biological control agents for plant diseases as *Lysobacter enzymogenes* based on phylogenetic analysis of 16s rdna, fatty acid composition and phenotypic characteristics. J. Appl. Microbiol..

[2] Folman L.B., Postma J., van Veen J.A. (2003). Characterisation of *Lysobacter enzymogenes* (Christensen and Cook 1978) strain 3.1T8, a powerful antagonist of fungal diseases of cucumber. Microbiol. Res..

[3] Giesler L.J., Yuen G.Y. (1998). Evaluation of *Stenotrophomonas maltophilia* strain C3 for biocontrol of brown patch disease. Crop Prot..

[4] Qian G.L., Hu B.S., Jiang Y.H., Liu F.Q. (2009). Identification and characterization of *Lysobacter enzymogenes* as a biological control agent against some fungal pathogens. Agric. Sci. China.

[5] Chen J., Moore W.H., Yuen G.Y., Kobayashi D., Caswell-Chen E.P. (2006). Influence of *Lysobacter enzymogenes* strain C3 on nematodes. J. Nematol..

[6] Kobayashi D.Y., Yuen G.Y. (2006). Molecular mechanisms of interactions between *Lysobacter enzymogenes* and plant pathogens. Phytopathology.

[7] Yuen G., Li S., Harris S., Yu F., Du L. (2005). An antibiotic produced by the biocontrol agent *Lysobacter enzymogenes* C3 inhibits fungal growth by blocking ceramide synthesis. Phytopathology.

[8] Yuen G.Y., Kobayashi D.K., Caswell-Chen E.P. (2006). Ecology and biological control of plant pathogens by *Lysobacter enzymogenes*. Phytopathology.

[9] Zhao Y., Qian G., Chen Y., Du L., Liu F. (2017). Transcriptional and antagonistic responses of biocontrol strain *Lysobacter enzymogenes* OH11 to the plant pathogenic oomycete pythium aphanidermatum. Front. Microbiol..

[10] Lou L.L., Qian G.L., Xie Y.X., Hang J.L., Chen H.T., Zaleta-Riyera K., Li Y.Y., Shen Y.M., Dussault P.H., Liu F.Q. (2011). Biosynthesis of hsaf, a tetramic acid-containing macrolactam from *Lysobacter enzymogenes*. J. Am. Chem. Soc..

[11] Jochum C.C., Osborne L.E., Yuen G.Y. (2006). Fusarium head blight biological control with *Lysobacter enzymogenes*. Biol. Control.

[12] Mathioni S.M., Patel N., Riddick B., Sweigard J.A., Czymmek K.J., Caplan J.L., Kunjeti S.G., Kunjeti S., Raman V., Hillman B.I. (2013). Transcriptomics of the rice blast fungus magnaporthe oryzae in response to the bacterial antagonist *Lysobacter enzymogenes* reveals candidate fungal defense response genes. PLoS ONE.

[13] Li S., Calvo A.M., Yuen G.Y., Du L., Harris S.D. (2009). Induction of cell wall thickening by the antifungal compound dihydromaltophilin disrupts fungal growth and is mediated by sphingolipid biosynthesis. J. Eukaryot. Microbiol..

[14] Ding Y.J., Li Z.Y., Li Y.Y., Lu C.H., Wang H.X., Shen Y.M., Du L.C. (2016). HSAF-induced antifungal effects in *Candida albicans* through ROS-mediated apoptosis. RSC Adv..

[15] Xu H.Y., Wang R.P., Zhao Y.Y., Fu Z.Q., Qian G.L., Liu F.Q. (2017). LesR is a novel upstream regulator that controls downstream Clp expression to modulate antibiotic HSAF biosynthesis and cell aggregation in *Lysobacter enzymogenes* OH11. Microb. Cell Fact..

[16] Wang R.P., Xu H.Y., Du L.C., Chou S.H., Liu H.X., Liu Y.Z., Liu F.Q., Qian G.L. (2016). A TonB-dependent receptor regulates antifungal HSAF biosynthesis in *Lysobacter*. Sci. Rep..

[17] Olson A.S., Chen H.T., Du L.C., Dussault P.H. (2015). Synthesis of a 2,4,6,8,10-dodecapentanoic acid thioester as a substrate for biosynthesis of heat stable antifungal factor (HSAF). RSC Adv..

[18] Li Y.Y., Huffman J., Li Y., Du L.C., Shen Y.M. (2012). 3-Hydroxylation of the polycyclic tetramate macrolactam in the biosynthesis of antifungal hsaf from *Lysobacter enzymogenes* C3. MedChemComm.

[19] Han Y., Wang Y., Tombosa S., Wright S., Huffman J., Yuen G., Qian G.L., Liu F.Q., Shen Y.M., Du L.C. (2015). Identification of a small molecule signaling factor that regulates the biosynthesis of the antifungal polycyclic tetramate macrolactam HSAF in *Lysobacter enzymogenes*. Appl. Microbiol. Biotechnol..

[20] Tanahashi M., Nakano T., Akamatsu H., Kodama M., Otani H., Osaki-Oka K. (2016). *Alternaria alternata* apple pathotype (*A. mali*) causes black spot of European pear. Eur. J. Plant Pathol..

[21] Duan S., Ma X., Chen W., Wan W., He Y., Ma X., Ma Y., Long N., Tan Y., Wang Y. (2016). Transcriptomic profile of tobacco in response to *Alternaria longipes* and *Alternaria alternata* infections. Sci. Rep..

[22] Lee S.S., Kawakita K., Tsuge T., Doke N. (1992). Stimulation of phospholipase-A_2_ activity in strawberry cells treated with AF-toxin-I produced by *Alternaria alternata* strawberry pathotype. Physiol. Mol. Plant Pathol..

[23] Tsuge T., Kobayashi H., Nishimura S. (1989). Organization of ribosomal RNA genes in *Alternaria alternata* japanese pear pathotype, a host-selective AK-toxin-producing fungus. Curr. Genet..

[24] Armitage A.D., Barbara D.J., Harrison R.J., Lane C.R., Sreenivasaprasad S., Woodhall J.W., Clarkson J.P. (2015). Discrete lineages within *Alternaria alternata* species group: Identification using new highly variable loci and support from morphological characters. Fungal Biol..

[25] Zeiner C.A., Purvine S.O., Zink E.M., Pasa-Tolic L., Chaput D.L., Haridas S., Wu S., LaButti K., Grigoriev I.V., Henrissat B. (2016). Comparative analysis of secretome profiles of manganese(II)-oxidizing ascomycete fungi. PLoS ONE.

[26] Li K.B. (2003). Clustalw-mpi: Clustalw analysis using distributed and parallel computing. Bioinformatics.

[27] Tamura K., Peterson D., Peterson N., Stecher G., Nei M., Kumar S. (2011). Mega5: Molecular evolutionary genetics analysis using maximum likelihood, evolutionary distance, and maximum parsimony methods. Mol. Biol. Evol..

[28] McCann M.P., Snetselaar K.M. (2008). A genome-based analysis of amino acid metabolism in the biotrophic plant pathogen *Ustilago maydis*. Fungal Genet. Biol..

[29] Li S., Du L., Yuen G., Harris S.D. (2006). Distinct ceramide synthases regulate polarized growth in the filamentous fungus *Aspergillus nidulans*. Mol. Biol. Cell.

[30] Wang Z., Shen Y. (2016). Antifungal compound honokiol triggers oxidative stress responsive signalling pathway and modulates central carbon metabolism. Mycology.

[31] Zhao Y., Paderu P., Park S., Dukhan A., Senter M., Perlin D.S. (2012). Expression turnover profiling to monitor the antifungal activities of amphotericin B, voriconazole, and micafungin against *Aspergillus fumigatus*. Antimicrob. Agents Chemother..

[32] Marioni J.C., Mason C.E., Mane S.M., Stephens M., Gilad Y. (2008). Rna-seq: An assessment of technical reproducibility and comparison with gene expression arrays. Genome Res..

[33] Wang M., Sun X., Yu D., Xu J., Chung K., Li H. (2016). Genomic and transcriptomic analyses of the tangerine pathotype of *Alternaria alternata* in response to oxidative stress. Sci. Rep..

[34] Muller C., Hjort C.M., Hansen K., Nielsen J. (2002). Altering the expression of two chitin synthase genes differentially affects the growth and morphology of *Aspergillus oryzae*. Microbiology.

[35] Feofilova E.P. (2010). The fungal cell wall: Modern concepts of its composition and biological function. Mikrobiologiia.

[36] Horiuchi H., Fujiwara M., Yamashita S., Ohta A., Takagi M. (1999). Proliferation of intrahyphal hyphae caused by disruption of *csm*A, which encodes a class V chitin synthase with a myosin motor-like domain in *Aspergillus nidulans*. J. Bacteriol..

[37] Liu J.H., Tang X., Wang H.Y., Balasubramanian M. (2000). Bgs2p, a 1,3-beta-glucan synthase subunit, is essential for maturation of ascospore wall in *Schizosaccharomyces pombe*. FEBS Lett..

[38] Lagorce A., Le Berre-Anton V., Aguilar-Uscanga B., Martin-Yken H., Dagkessamanskaia A., Francois J. (2002). Involvement of *GFA1*, which encodes glutamine-fructose-6-phosphate amidotransferase, in the activation of the chitin synthesis pathway in response to cell-wall defects in *Saccharomyces cerevisiae*. FEBS J..

[39] Garcia A.B., Vinuela-Prieto J.M., Lopez-Gonzalez L., Candel F.J. (2017). Correlation between resistance mechanisms in *Staphylococcus aureus* and cell wall and septum thickening. Infect. Drug Resist..

[40] Zhao Y., Song D., Sun J., Li L. (2013). Populus endo-beta-mannanase ptrman6 plays a role in coordinating cell wall remodeling with suppression of secondary wall thickening through generation of oligosaccharide signals. Plant J. Cell Mol. Biol..

[41] Hyo Y., Yamada S., Fukutsuji K., Harada T. (2013). Thickening of the cell wall in macrolide-resistant *Staphylococcus aureus*. Med. Mol. Morphol..

[42] He F., Zhang X., Mafurah J.J., Zhang M., Qian G., Wang R., Safdar A., Yang X., Liu F., Dou D. (2016). The transcription factor vpcrz1 is required for fruiting body formation and pathogenicity in *Valsa pyri*. Microb. Pathog..

[43] Tao L., Zhang Y.L., Fan S.R., Nobile C.J., Guan G.B., Huang G.H. (2017). Integration of the tricarboxylic acid (TCA) cycle with camp signaling and Sfl2 pathways in the regulation of CO_2_ sensing and hyphal development in *Candida albicans*. PLos Genet..

[44] Paumen M.B., Ishida Y., Muramatsu M., Yamamoto M., Honjo T. (1997). Inhibition of carnitine palmitoyltransferase I augments sphingolipid synthesis and palmitate-induced apoptosis. J. Biol. Chem..

[45] Krown K.A., Page M.T., Nguyen C., Zechner D., Gutierrez V., Comstock K.L., Glembotski C.C., Quintana P.J., Sabbadini R.A. (1996). Tumor necrosis factor alpha-induced apoptosis in cardiac myocytes. Involvement of the sphingolipid signaling cascade in cardiac cell death. J. Clin. Investig..

[46] Young M.M., Kester M., Wang H.G. (2013). Sphingolipids: Regulators of crosstalk between apoptosis and autophagy. J. Lipid Res..

[47] Yamaji-Hasegawa A., Takahashi A., Tetsuka Y., Senoh Y., Kobayashi T. (2005). Fungal metabolite sulfamisterin suppresses sphingolipid synthesis through inhibition of serine palmitoyltransferase. Biochemistry.

[48] Hwang S., Gustafsson H.T., O’Sullivan C., Bisceglia G., Huang X.H., Klose C., Schevchenko A., Dickson R.C., Cavaliere P., Dephoure N. (2017). Serine-dependent sphingolipid synthesis is a metabolic liability of aneuploid cells. Cell Rep..

[49] Freitas F.Z., de Paula R.M., Barbosa L.C., Terenzi H.F., Bertolini M.C. (2010). Camp signaling pathway controls glycogen metabolism in neurospora crassa by regulating the glycogen synthase gene expression and phosphorylation. Fungal Genet. Biol..

[50] Altiok S., Xu M., Spiegelman B.M. (1997). Ppargamma induces cell cycle withdrawal: Inhibition of E2F/DP DNA-binding activity via down-regulation of PP2A. Genes Dev..

[51] Ruvolo P.P., Deng X., Ito T., Carr B.K., May W.S. (1999). Ceramide induces Bcl2 dephosphorylation via a mechanism involving mitochondrial PP2A. J. Biol. Chem..

[52] Kurimchak A., Grana X. (2012). PP2A holoenzymes negatively and positively regulate cell cycle progression by dephosphorylating pocket proteins and multiple CDK substrates. Gene.

[53] Cammisotto P.G., Londono I., Gingras D., Bendayan M. (2008). Control of glycogen synthase through ADIPOR1-AMPK pathway in renal distal tubules of normal and diabetic rats. Am. J. Phys.-Renal Physiol..

[54] Park M., Youn B., Zheng X.L., Wu D., Xu A., Sweeney G. (2011). Globular adiponectin, acting via AdipoR1/APPL1, protects H9c2 cells from hypoxia/reoxygenation-induced apoptosis. PLoS ONE.

[55] Song J., Choi S.M., Whitcomb D.J., Kim B.C. (2017). Adiponectin controls the apoptosis and the expression of tight junction proteins in brain endothelial cells through AdipoR1 under beta amyloid toxicity. Cell Death Dis..

[56] Tian X.Q., Yang Y.J., Li Q., Huang P.S., Li X.D., Jin C., Qi K., Jiang L.P., Chen G.H. (2016). Globular adiponectin inhibits the apoptosis of mesenchymal stem cells induced by hypoxia and serum deprivation via the AdipoR1-mediated pathway. Cell. Physiol. Biochem..

[57] Liu G.Z., Liang B., Lau W.B., Wang Y., Zhao J., Li R., Wang X., Yuan Y., Lopez B.L., Christopher T.A. (2015). High glucose/high lipids impair vascular adiponectin function via inhibition of caveolin-1/AdipoR1 signalsome formation. Free Radic. Biol. Med..

[58] Ishikawa M., Kitayama J., Yamauchi T., Kadowaki T., Maki T., Miyato H., Yamashita H., Nagawa H. (2007). Adiponectin inhibits the growth and peritoneal metastasis of gastric cancer through its specific membrane receptors AdipoR1 and AdipoR2. Cancer Sci..

[59] Qian G., Wang Y., Liu Y., Xu F., He Y.W., Du L., Venturi V., Fan J., Hu B., Liu F. (2013). *Lysobacter enzymogenes* uses two distinct cell-cell signaling systems for differential regulation of secondary-metabolite biosynthesis and colony morphology. Appl. Environ. Microbiol..

[60] Takaoka S., Kurata M., Harimoto Y., Hatta R., Yamamoto M., Akimitsu K., Tsuge T. (2014). Complex regulation of secondary metabolism controlling pathogenicity in the phytopathogenic fungus *Alternaria alternata*. New Phytol..

[61] Finn R.D., Clements J., Arndt W., Miller B.L., Wheeler T.J., Schreiber F., Bateman A., Eddy S.R. (2015). Hmmer web server: 2015 update. Nucleic Acids Res..

[62] Langmead B., Salzberg S.L. (2012). Fast gapped-read alignment with bowtie 2. Nat. Methods.

[63] Kim D., Pertea G., Trapnell C., Pimentel H., Kelley R., Salzberg S.L. (2013). TopHat2: Accurate alignment of transcriptomes in the presence of insertions, deletions and gene fusions. Genome Biol..

[64] Anders S., Pyl P.T., Huber W. (2015). HTSeq—A python framework to work with high-throughput sequencing data. Bioinformatics.

[65] Anders S., Huber W. (2010). Differential expression analysis for sequence count data. Genome Biol..

[66] Li B., Dewey C.N. (2011). RSEM: Accurate transcript quantification from RNA-Seq data with or without a reference genome. BMC Bioinform..

[67] Anders S., Huber W. (2013). Differential expression of RNA-Seq data at the gene level—The DESeq package. EMBL.

[68] Young M.D., Wakefield M.J., Smyth G.K., Oshlack A. (2010). Gene ontology analysis for RNA-Seq: Accounting for selection bias. Genome Biol..

[69] Mao X., Cai T., Olyarchuk J.G., Wei L. (2005). Automated genome annotation and pathway identification using the KEGG orthology (KO) as a controlled vocabulary. Bioinformatics.

[70] Altschul S.F., Madden T.L., Schaffer A.A., Zhang J., Zhang Z., Miller W., Lipman D.J. (1997). Gapped blast and psi-blast: A new generation of protein database search programs. Nucleic Acids Res..

