# Supplementary material for: Transcriptomics Analysis of the Chinese Pear Pathotype of Alternaria alternata Gives Insights into Novel Mechanisms of HSAF Antifungal Activities
Source: Int J Mol Sci. 2018 Jun 22;19(7):1841. doi: 10.3390/ijms19071841 (PMC6073358; doi:10.3390/ijms19071841)
Supplement: Supplementary file 1 [file ijms-19-01841-s001.zip › Table S1.docx]

| **Table S1 Qualify analyses of RNA-seq** | |  |  |  |  |  |
| --- | --- | --- | --- | --- | --- | --- |
|  | Samples | | | | | |
|  | HSAF_1 | HSAF_2 | HSAF_3 | CK_1 | CK_2 | CK_3 |
| Raw reads | 26516520 | 29330688 | 26604218 | 25828242 | 29356510 | 24164072 |
| Clean reads | 24715792 | 27413170 | 25172530 | 24233886 | 27648044 | 22507840 |
| Clean bases(G) | 3.73 | 4.13 | 3.8 | 3.65 | 4.17 | 3.39 |
| Error rate(%) | 0.03 | 0.03 | 0.02 | 0.03 | 0.02 | 0.02 |
| Q20(%) | 94.73 | 94.49 | 95.14 | 94.59 | 95.24 | 94.9 |
| Q30(%) | 88.01 | 87.49 | 88.77 | 87.76 | 89.03 | 88.3 |
| GC content(%) | 54.06 | 54.38 | 54.35 | 54.1 | 54.41 | 54.3 |
| Total reads | 24715792 | 27413170 | 25172530 | 24233886 | 27648044 | 22507840 |
| Total mapped | 20631010 (83.47%) | 22801963 (83.18%) | 21126322 (83.93%) | 19974117 (82.42%) | 22864552 (82.7%) | 18595565 (82.62%) |
| Multiple mapped | 81598 (0.33%) | 74355 (0.27%) | 80481 (0.32%) | 56760 (0.23%) | 69532 (0.25%) | 51798 (0.23%) |
| Uniquely mapped | 20549412 (83.14%) | 22727608 (82.91%) | 21045841 (83.61%) | 19917357 (82.19%) | 22795020 (82.45%) | 18543767 (82.39%) |
| Read-1 | 10636001 (43.03%) | 11773751 (42.95%) | 10833429 (43.04%) | 10326147 (42.61%) | 11731768 (42.43%) | 9563596 (42.49%) |
| Read-2 | 9913411 (40.11%) | 10953857 (39.96%) | 10212412 (40.57%) | 9591210 (39.58%) | 11063252 (40.01%) | 8980171 (39.9%) |
| Reads map to '+' | 10275267 (41.57%) | 11358257 (41.43%) | 10522042 (41.8%) | 9942991 (41.03%) | 11381390 (41.17%) | 9259722 (41.14%) |
| Reads map to '-' | 10274145 (41.57%) | 11369351 (41.47%) | 10523799 (41.81%) | 9974366 (41.16%) | 11413630 (41.28%) | 9284045 (41.25%) |
| Non-splice reads | 15953270 (64.55%) | 17631400 (64.32%) | 16321775 (64.84%) | 15597512 (64.36%) | 17832268 (64.5%) | 14520943 (64.52%) |
| Splice reads | 4596142 (18.6%) | 5096208 (18.59%) | 4724066 (18.77%) | 4319845 (17.83%) | 4962752 (17.95%) | 4022824 (17.87%) |
