# Supplementary material for: Transcriptomics Analysis of the Chinese Pear Pathotype of Alternaria alternata Gives Insights into Novel Mechanisms of HSAF Antifungal Activities
Source: Int J Mol Sci. 2018 Jun 22;19(7):1841. doi: 10.3390/ijms19071841 (PMC6073358; doi:10.3390/ijms19071841)
Supplement: Supplementary file 1 [file ijms-19-01841-s001.zip › Table S12.docx]

| **Table S12 Primers for RT-qPCR** | |
| --- | --- |
| Gene ID | primer sequence |
| CC77DRAFT_1023491 | F:5' TTCTTCGTCTTCATGTGCGTCT 3' |
|  | R:5' TGTATTGTTGCGTTTCCCAGTT 3' |
| CC77DRAFT_1086185 | F:5' CAATGCTTGGTTCAGCGACTA 3' |
|  | R:5' GAGCAACGCCATCTTACTTCC 3' |
| CC77DRAFT_926244 | F: 5' TTACTCGGCAACCAGACGC 3' |
|  | R:5' AAGCCCTCGGCAGCATTT 3' |
| CC77DRAFT_960140 | F:5' CGGCAGAAATTATCAACACGC 3' |
|  | R:5' TTCCAGTCCCAACGAGCAA 3' |
| CC77DRAFT_973065 | F:5' GGCAACGATATTCCCATCCA 3' |
|  | R:5' CACCAGCATACACGAGACCAAA 3' |
| CC77DRAFT_134151 | F:5' GTTCCGTGCTTTTCTCCCG 3' |
|  | R:5' GGTCGTAGCCTGTCGTATCTCA 3' |
| CC77DRAFT_389685 | F:5' TAGATGGCGAGCACCACAAC 3' |
|  | R:5' CGACAATGACCCACCCGTA 3' |
| CC77DRAFT_741026 | F:5' TCGCCACTATCCGTGCTATG 3' |
|  | R:5' CCGACCAGTAATGTTCCTCCA 3' |
| CC77DRAFT_487727 | F:5' ACAAACCCAGAACGCAGCAC 3' |
|  | R:5' GAAGAAAAGGGCAAGAATCAGC 3' |
| CC77DRAFT_482709 | F:5' AGCCATACAAGACGAGCGACA 3' |
|  | R:5' TCACAACCAGGATGACCGAGA 3' |
